# Use of oral neuromodulators in chronic pelvic painNumber 8 – 2025

**DOI:** 10.61622/rbgo/2025FPS8

**Published:** 2025-10-29

**Authors:** Omero Benedicto Poli, Julio Cesar Rosa e Silva, Carlos Alberto Petta, Carlos Augusto Pires Costa Lino, Eduardo Schor, Helizabet Salomão Abdalla Ayroza Ribeiro, João Nogueira, João Sabino Lahorgue da Cunha, Marcia Cristina França, Márcia Mendonça Carneiro, Marco Aurélio Pinho de Oliveira, Marcos Tcherniakovsky, Maurício Simões Abrão, Raquel Papandreus Dib, Ricardo de Almeida Quintairos, Sergio Podgaec, Sidney Pearce

**Affiliations:** Departamento de Ginecologia e Obstetrícia Faculdade de Medicina de Ribeirão Preto Universidade de São Paulo Ribeirão Preto SP Brazil Departamento de Ginecologia e Obstetrícia, Faculdade de Medicina de Ribeirão Preto, Universidade de São Paulo, Ribeirão Preto, SP, Brazil.; Departamento de Ginecologia e Obstetrícia Faculdade de Medicina de Ribeirão Preto Universidade de São Paulo Ribeirão Preto SP Brazil Departamento de Ginecologia e Obstetrícia, Faculdade de Medicina de Ribeirão Preto, Universidade de São Paulo, Ribeirão Preto, SP, Brazil.; Universidade Estadual de Campinas Campinas SP Brazil Universidade Estadual de Campinas, Campinas, SP, Brazil.; Clínica Fertilidade & Vida Campinas SP Brazil Clínica Fertilidade & Vida, Campinas, SP, Brazil.; Serviço de Reprodução Assistida Hospital Sírio-Libanês São Paulo SP Brazil Serviço de Reprodução Assistida, Hospital Sírio-Libanês, São Paulo, SP, Brazil.; Hospital Aliança Salvador BA Brazil Hospital Aliança, Salvador, BA, Brazil.; Instituto de Perinatologia da Bahia Salvador BA Brazil Instituto de Perinatologia da Bahia, Salvador, BA, Brazil.; Escola Paulista de Medicina Universidade Federal de São Paulo São Paulo SP Brazil Escola Paulista de Medicina, Universidade Federal de São Paulo, São Paulo, SP, Brazil.; Faculdade de Ciências Médicas Santa Casa de São Paulo São Paulo SP Brazil Faculdade de Ciências Médicas, Santa Casa de São Paulo, São Paulo, SP, Brazil.; Universidade Federal do Maranhão São Luís MA Brazil Universidade Federal do Maranhão, São Luís, MA, Brazil.; Departamento de Ginecologia e Obstetrícia Universidade Federal do Rio Grande do Sul Porto Alegre RS Brazil Departamento de Ginecologia e Obstetrícia, Universidade Federal do Rio Grande do Sul, Porto Alegre, RS, Brazil.; Departamento de Ginecologia e Obstetrícia Faculdade de Medicina Universidade Federal de Minas Gerais Belo Horizonte MG Brazil Departamento de Ginecologia e Obstetrícia, Faculdade de Medicina, Universidade Federal de Minas Gerais, Belo Horizonte, MG, Brazil.; Departamento de Ginecologia e Obstetrícia Faculdade de Medicina Universidade Federal de Minas Gerais Belo Horizonte MG Brazil Departamento de Ginecologia e Obstetrícia, Faculdade de Medicina, Universidade Federal de Minas Gerais, Belo Horizonte, MG, Brazil.; Faculdade de Ciências Médicas Universidade do Estado do Rio de Janeiro Rio de Janeiro RJ Brazil Faculdade de Ciências Médicas, Universidade do Estado do Rio de Janeiro, Rio de Janeiro, RJ, Brazil.; Setor de Videoendoscopia Ginecológica e Endometriose Faculdade de Medicina do ABC Santo André SP Brazil Setor de Videoendoscopia Ginecológica e Endometriose, Faculdade de Medicina do ABC, Santo André, SP, Brazil.; Divisão de Ginecologia Hospital Beneficência Portuguesa de São Paulo São Paulo SP Brazil Divisão de Ginecologia, Hospital Beneficência Portuguesa de São Paulo, São Paulo, SP, Brazil.; Departamento de Ginecologia e Obstetrícia Faculdade de Medicina Universidade de São Paulo São Paulo SP Brazil Departamento de Ginecologia e Obstetrícia, Faculdade de Medicina, Universidade de São Paulo, São Paulo, SP, Brazil.; Universidade Federal de Ciências da Saúde de Porto Alegre Porto Alegre RS Brazil Universidade Federal de Ciências da Saúde de Porto Alegre, Porto Alegre, RS, Brazil.; Núcleo de Endometriose Hospital Porto Dias Belém PA Brazil Núcleo de Endometriose, Hospital Porto Dias, Belém, PA, Brazil.; Faculdade de Medicina Universidade de São Paulo São Paulo SP Brazil Disciplina de Obstetrícia e Ginecologia, Faculdade de Medicina, Universidade de São Paulo, São Paulo, SP, Brazil.; Hospital Israelita Albert Einstein São Paulo SP Brazil Hospital Israelita Albert Einstein, São Paulo, SP, Brazil.; Centro Universitário Christus Fortaleza CE Brazil Centro Universitário Christus, Fortaleza, CE, Brazil.

## Abstract

•Chronic pelvic pain is a common and complex condition that significantly affects women’s quality of life.

•Neuropathic pain and nociplastic pain are important components in the pain picture of these patients and should be considered in clinical treatment.

•Oral neuromodulators, antidepressants and anticonvulsants for the control of neuropathic and nociplastic pain should be present in the therapeutic arsenal of the gynecologist who treats patients with chronic pelvic pain.

•Pregabalin is the medication with the best pharmacokinetic profile; nortriptyline has the best adverse effects profile; duloxetine is the most widely used and has the lowest risks; and venlafaxine should be used as a second-line inhibitor.

•Although the drug classes can be combined to reduce the total doses and minimize side effects, maximizing the analgesic effect, monotherapies are recommended as the first line to avoid polypharmacy.

**Recommendations**

- We recommend the following order of priority as first-line treatment for neuropathic pain: 1) gabapentinoids; 2) tricyclic antidepressants; 3) serotonin and norepinephrine reuptake inhibitor (SNRI) antidepressants.
- We recommend the following order of priority as first-line treatment for nociplastic pain: 1) SNRI antidepressants; 2) gabapentinoids; 3) tricyclic antidepressants.
- The use of cannabinoids should be restricted to academic research settings adhering to rigorous scientific protocols until robust scientific evidence is published.
- There is no time limit for maintaining treatment. As a rule, we recommend a period of at least six months and a maximum of 12 months. After that, and in accordance with the patient and other coping strategies, gradual discontinuation should be discussed;
- The following should always be kept in mind: 1) achieving the lowest possible pain scores; 2) using the smallest possible amount of medication; 3) using the lowest possible dose of each medication; 4) minimizing side effects; 5) maximizing patient adherence; 6) reinforcing coping mechanisms and positive thinking.

## Background

Chronic pelvic pain is a common and complex condition that can negatively affect people’s quality of life. Various associated diseases can lead to symptoms through diverse mechanisms. In clinical practice, in addition to seeking a specific diagnosis, it is essential to typify the pain perceived by the patient. This is crucial in planning the therapeutic approach and consequently, achieving better medium and long-term outcomes.^(1)^

Pain can be simplified and classified as nociceptive (also known as inflammatory), neuropathic and nociplastic. Other categories can be listed, but are beyond the scope of this statement. In the first case, it is triggered predominantly by peripheral mechanisms directly induced by the underlying disease(s). Thus, treating the underlying disease and using adjuvant analgesics from the non-steroidal anti-inflammatory drug (NSAID) class is generally effective for curing and/or controlling pain for a relatively long period.^(2)^ In the case of neuropathic pain, the primary condition may also be directly responsible for the neurological impairment. Thus, its treatment often results in substantial improvement in pain, but neurological damage often persists and personalized adjuvant treatment must be instituted. In this case, NSAIDs are not effective and may even lead to an increase in the perception of pain. Gabapentinoids have proven to be the most effective drugs in relieving this symptom, but antidepressants have excellent results that have been known for a long time.

As should be evident from everyone’s clinical experience, specific treatment of these “primary” diseases does not always result in the cure or prolonged control of painful symptoms. This is due, at least in part, to the involvement of the central nervous system (CNS) in the pathophysiology of chronic pain.^(3)^ The pain resulting from this condition is called nociplastic and is triggered by changes in the CNS, the main one being central sensitization (CS). Briefly, CS is a process in which the individual becomes more sensitive to pain due to the reduction or loss of effectiveness of the inhibitory modulation system. Perhaps the most curious and particular thing about this condition is that it is the primary cause of the perpetuation of chronic pain. In other words, in this case, the painful symptom does not depend on a possible primary triggering cause. Even if it is completely eliminated, the symptoms persist.^(4)^ The study of nociplastic pain has been the focus in the investigation of processes that trigger and maintain chronic pain. Clinically, it has been associated with symptoms such as changes in mood, memory and sleep patterns, and fatigue.^(5)^

The medications we will discuss in this statement are part of the arsenal we can use to provide symptomatic relief to patients and have the greatest scientific basis in the current literature.

Before delving into a specific discussion on the topic, it is important to emphasize the vast and rapidly expanding field of pain pathophysiology. It is essential that we have the curiosity and responsibility to always keep ourselves updated. It is also important to emphasize two additional points. The first is that we do not intend to exhaust the topic and/or present all the possibilities of adjuvant treatments. We only present considerations of the medications that have proven to be the most effective in controlling chronic pelvic pain, whether as adjuvant or first-line treatment, or that have had increasing media appeal. We will focus on three classes: antidepressants, gabapentinoids and cannabinoids. The second point are the several types of non-drug treatments included in the therapeutic arsenal that play an important role in the lasting relief of symptoms. These modalities should not be underestimated and be used as adjuvant measures whenever possible.

### Antidepressants. When and how to use them?

Antidepressants have been used for many decades, including for the treatment of chronic pain. However, not all classes of medication are effective. We will focus on tricyclics and dual selective antidepressants, which make up the group with the best results.^(6)^ Selective serotonin reuptake inhibitors (SSRIs) have not been consistently effective^(7)^ and have shown inferior results than tricyclics.^(8)^ For this reason, they will not be included in this statement, but may be considered in special situations by specialists in the field. Examples of these medications include paroxetine, fluoxetine, sertraline, citalopram and escitalopram.

### Tricyclic antidepressants

#### Description

Tricyclic antidepressants (TCAs) have been used for decades to treat depression and various types of pain (off-label). These psychotropic drugs were launched on the market in the 1960s. The best-known substances are imipramine, amitriptyline, and nortriptyline, but as the first has not shown uniform and robust results in the treatment of chronic pain, we will focus on the use of amitriptyline and nortriptyline.

#### Mechanisms of action

Tricyclic antidepressants exert their analgesic effects through multiple mechanisms:

1. Inhibition of serotonin and norepinephrine reuptake, leading to increased synaptic levels of these neurotransmitters, which are important in the inhibitory modulation of pain perception;
2. Blockade of sodium channels in peripheral nerves, reducing the transmission of pain signals along nerve fibers. This characteristic is especially relevant in the control of neuropathic pain; and
3. Modulation of the N-methyl-D-aspartate (NMDA) receptor, which plays a role in CS and the perception of chronic pain, which is attributed to the potential for controlling nociplastic pain.

#### Efficacy in chronic pain

Numerous clinical trials and observational studies have demonstrated the efficacy of TCAs in the management of chronic pain conditions. Currently, their main application is in neuropathic pain and nociplastic pain. This class is effective in the treatment of neuropathic pain, such as that associated with diabetic neuropathy, postherpetic neuralgia, and peripheral neuropathy.^(9)^ Conditions such as fibromyalgia and chronic pelvic pain are often associated with a significant nociplastic component, and TCAs have also shown to be effective in these conditions. Nortriptyline is often preferred in these cases due to its favorable side effect profile. Despite its proven effect, the Food and Drug Administration (FDA) has not approved the drug for the treatment of pain, meaning its use in the United States is off-label.

#### Dosage recommendations

Due to the high prevalence of side effects and the consequent difficulty in adherence, it is recommended to start with the lowest possible doses, followed by a progressive and supervised increase. There is still a gap in the literature regarding the ideal dosage, duration of treatment and long-term results associated with the use of TCAs in pain management. Clinical improvement is usually noticeable after one or two weeks of starting use. However, it can take an average of six weeks or more to obtain a significant result (reduction of at least 30% in initial pain scores).

Before starting the prescription, the professional must bear in mind that the goal is to maintain the lowest possible effective dose. Based on this, the recommendations are as follows:

Amitriptyline and nortriptyline: the starting dose should be as low as possible (between 10-25 mg/day), followed by weekly or biweekly assessment with progressive and additional increases of 25 mg/day. Once the dose of 75-100 mg/day has been reached, maintaining this for a longer period (approximately four weeks) is prudent before increasing further. The maximum recommended dose of amitriptyline is 300 mg/day and that of nortriptyline is 150 mg/day. Doses above 150 mg/day have been associated with little improvement in the control of chronic pelvic pain. Once the therapeutic dose has been established, we recommend maintaining it for 6-12 months, depending on the individual progress of each patient and other pain control measures or coping mechanisms incorporated. Abrupt discontinuation of this medication is strongly discouraged.

#### Side effects

Although TCAs can be effective in managing pain, they are not free from side effects, which are mostly due to their anticholinergic action, but not exclusively. The most common side effects include dry mouth, constipation, sedation, weight gain, reduced sexual desire, blurred vision, tremor, sweating, nausea and tachycardia. In older adults, TCAs may increase the risk of falls and cognitive impairment. Less common side effects include orthostatic hypotension, arrhythmias, urinary retention, dry eyes, and memory problems. Therefore, careful patient selection and monitoring are essential when using TCAs for pain management.

#### Risks and precautions

1. Suicidal thoughts: as with many antidepressant medications, some people, especially children, adolescents, and young adults, may experience an increase in suicidal thoughts or behaviors when taking TCAs. Close monitoring and regular communication with a healthcare professional are crucial during the first few weeks of treatment.
2. Serotonin syndrome: this is characterized by anxiety, irritability, muscle twitching, confusion and hallucinations, tremors and chills, nausea and diarrhea, increased blood pressure and heart rate, increased reflexes, and dilated pupils. The triggering may be associated not only with the use of high doses, but also with the concomitant use of other antidepressants, triptans for migraine (e.g., sumatriptan), opioids (codeine, tramadol, meperidine, etc.), antiemetics (metoclopramide, ondansetron), anticonvulsants (carbamazepine, valproic acid), erythromycin, ciprofloxacin, fluconazole, ritonavir, cocaine, amphetamines, LSD, ecstasy, tryptophan, St. John’s wort, and ginseng.^(10)^
3. Withdrawal symptoms: abrupt discontinuation of TCAs may lead to withdrawal symptoms, including nausea, headache, and mood swings. Gradually reducing the medication under the guidance of a healthcare professional may help mitigate the effects of withdrawal.

#### Drug interactions

1. Monoamine oxidase inhibitors (MAOIs): combining TCAs with MAOIs can lead to a potentially life-threatening condition called serotonin syndrome. There must be a significant time interval (usually two weeks or more) between discontinuing an MAOI and starting a TCA.
2. Serotonin: combining TCAs with other serotonergic drugs, such as selective serotonin reuptake inhibitors (SSRIs) or serotonin-norepinephrine reuptake inhibitors (SNRIs), may increase the risk of serotonin syndrome.
3. Antipsychotics: some antipsychotic drugs, especially those with anticholinergic effects such as chlorpromazine, may interact with TCAs and significantly increase the risk of side effects.
4. Anticholinergics: concomitant use with antihistamines or antiparkinsonian drugs may also increase the risk of side effects.
5. Antiarrhythmics: TCAs can affect cardiac conduction, so combining them with certain antiarrhythmic medications may increase the risk of arrhythmias.
6. Anticoagulants: there may be an increased risk of bleeding due to the antiplatelet activity of TCAs.
7. Antihypertensives: TCAs may interact with some medications in the beta-blocker, calcium channel blocker, and clonidine classes, potentially causing additive or antagonistic effects on blood pressure.
8. Interactions with cytochrome P450 (CYP450) enzymes: TCAs may interact with the CYP450 enzyme system, potentially affecting the metabolism of other medications.

## Selective serotonin and norepinephrine reuptake inhibitors

### Description

Selective serotonin and norepinephrine reuptake inhibitors, also called “duals”, initially gained notoriety in the treatment of depression due to their excellent results, coupled with a better side effect profile than tricyclics. Their recognition as a class of drugs with analgesic properties is mainly due, but not only, to their effect as a modulator of norepinephrine reuptake, which is essential in modulating pain in humans. They have been on the world market since the 1990s. The most widely known substances are: duloxetine, venlafaxine and desvenlafaxine.

### Mechanisms of action

Serotonin-norepinephrine reuptake inhibitors such as duloxetine and venlafaxine, modulate neurotransmitter levels in the brain and spinal cord through a main mechanism,^(11)^ the inhibition of serotonin and norepinephrine reuptake in the synaptic cleft.

### Efficacy in chronic pain

Serotonin-norepinephrine reuptake inhibitors have demonstrated efficacy in the treatment of several chronic pain conditions, particularly those including neuropathic and nociplastic components such as fibromyalgia, diabetic neuropathy, peripheral neuropathy, and chronic musculoskeletal pain. Duloxetine, in particular, is FDA-approved for these indications and is the only one with good evidence in the literature for significant pain reduction and proven improvement in quality of life in clinical trials.^(12,13)^

### Dosage recommendations

Although the side effect profile is slightly better compared to the use of tricyclics, we recommend starting with a low dose and gradually increasing it to reduce the rate of adverse effects and, consequently, avoid loss of adherence. There is still a gap in the literature regarding optimal dosage, duration of treatment, and long-term outcomes associated with the use of SNRIs in pain management. Clinical improvement may be noticeable from the first 24 hours of use, but generally after the first week. On average, it takes about 30-40 days to obtain a significant result (at least a 30% reduction in initial pain scores).

Before starting the prescription, the professional should keep in mind that the goal is to maintain the lowest possible effective dose. Based on this, the recommendations are as follows:

Duloxetine: the starting dose should be as low as possible (about 20 mg/day), followed by a weekly assessment. Any dose increases should be progressive and, preferably, 20 mg/day each week. Once the dose of 60 mg/day is reached, maintaining this for a longer period (about 2-3 weeks) is prudent before further progressions. The maximum recommended dose is 120 mg/day. Doses higher than 60 mg/day have not shown significant additional benefits and safety has not been adequately evaluated.

Venlafaxine: the starting dose should be as low as possible (about 37.5 mg/day), followed by a weekly assessment. Any dose increases should be progressive and preferably 37.5 mg/day each week. Once the dose of 150 mg/day has been reached, maintaining this for a longer period (approximately 2-3 weeks) is prudent before increasing further. Doses higher than 225 mg/day are not usual to ensure efficacy. Doses higher than 375 mg/day should not be prescribed.

Desvenlafaxine: the usual starting dose is 50 mg/day. Progression should occur after two weeks, but doses higher than 50 mg/day have not been shown to provide additional benefits for pain control. More recently, the recommended treatment has been to start with just 25 mg/day, with possible progression after two weeks. Doses higher than 400 mg/day should not be prescribed.

### Side effects

Although generally well tolerated, SNRIs can cause side effects, including nausea, headache, dizziness, dry mouth, drowsiness or insomnia, sedation, excessive sweating, reduced sexual desire, and difficulty achieving orgasm. Increased blood pressure is uncommon, but is especially associated with the use of venlafaxine at higher doses. Regular monitoring of blood pressure may be necessary. Less commonly, it is associated with serotonin syndrome, which has been described previously.

### Risks and precautions

1. Suicidal thoughts: some people, especially children, adolescents, and young adults, may experience an increase in suicidal thoughts or behavior when taking SSRIs. Close monitoring and regular communication with a healthcare professional are crucial during the first few weeks of treatment.
2. Risk of bleeding: SSRIs may increase the risk of bleeding, especially when combined with anticoagulant or antiplatelet medications. Use caution if you are taking these medications at the same time.
3. Serotonin syndrome: this is less commonly seen and has been described previously. Patients should be informed of the risk and instructed to recognize the signs and symptoms.
4. Withdrawal symptoms: Stopping SSRIs abruptly can lead to withdrawal symptoms, such as dizziness, nausea, headache, irritability, and mood swings. Gradually reducing the medication under the guidance of a healthcare professional can help mitigate the effects of withdrawal.

### Drug interactions

1. MAOIs: Combining SSRIs with MAOIs can lead to serotonin syndrome. There should be a significant interval (usually two weeks or more) between stopping an MAOI and starting an SSRI.
2. Serotonin: combining SSRIs with other serotonergic medications, such as SSRIs or triptans (used for migraines), can also increase the risk of serotonin syndrome.
3. Anticoagulants and antiplatelet agents: SSRIs may increase the risk of bleeding when taken with anticoagulant drugs, such as warfarin, or antiplatelet agents, such as aspirin.
4. Antihypertensives: TCAs may interact with some drugs in the beta-blocker, calcium channel blocker and clonidine classes, which may cause additive or antagonistic effects on blood pressure.
5. CYP450 enzyme inhibitors: some drugs that inhibit the CYP450 enzyme system may increase blood levels of SSRIs, which may lead to side effects or toxicity.

## Gabapentinoids – when and how to use them?

### Description

Gabapentinoids are drugs in the anticonvulsant class. They are analogues of gamma-aminobutyric acid (GABA), which is one of the main inhibitory neurotransmitters in the CNS. The substances that represent this class are gabapentin and pregabalin. As mentioned, they were initially developed for the treatment of epilepsy in the 1990s, but were quickly incorporated as drugs for the treatment of pain at the beginning of the century, especially neuropathic pain. There is currently extensive evidence to support its use as the first line of treatment for this condition.^(14,15)^ Despite being newer and having fewer studies in the literature, pregabalin appears to have the same efficacy.^(16)^ It is important to emphasize that its indiscriminate use without clear criteria for the treatment of chronic pelvic pain in women is not supported by the literature.^(17)^

### Mechanisms of action

Gabapentinoids exert their analgesic effects through the following mechanisms:^(18)^

1. Inhibition of calcium channels: gabapentinoids bind primarily to the α2δ subunit of voltage-gated calcium channels in the CNS, reducing calcium entry into neurons. This action decreases the release of neurotransmitters and neuronal excitability, contributing to pain modulation.
2. GABAergic activity: gabapentinoids can increase GABA neurotransmission, which can inhibit excessive signaling in pain pathways.

### Efficacy in chronic pain

Clinical trials and real-world evidence support the use of gabapentinoids in the management of chronic pain conditions, including diabetic neuropathy, postherpetic neuralgia, and chronic low back pain. Pregabalin is FDA-approved for these indications and has demonstrated efficacy in reducing pain and improving quality of life.

### Dosage recommendations

The side effect profile is better than that of antidepressants, but we also recommend starting with a low dose and gradually increasing it to reduce the rate of side effects and, consequently, avoid loss of adherence. While gabapentin has a nonlinear pharmacokinetic profile, pregabalin has a linear relationship between the dose used and the plasma concentration. This particular pharmacodynamic feature makes pregabalin more comfortable to use (twice a day, instead of three times a day as with gabapentin) and has effective results observed more quickly (after one day of use, compared to 9-10 for gabapentin). On average, it takes about 5-6 weeks to obtain a significant result (at least a 30% reduction in initial pain scores).

Before starting the prescription, the professional should keep in mind the goal to maintain the lowest possible effective dose. Based on this, the recommendations are as follows:

Gabapentin: the initial dose is 900 mg/day divided into three doses (three 300 mg tablets/day). It is usual to follow a progressive schedule: 300 mg on day one, 600 mg on day two, and 900 mg from day three onwards. From then on, it is possible to progress in increments of 300 mg/day every 4-5 days up to a total dose of 1,800 mg/day. Data associated with significant improvement above this dose are still lacking. The dose should not exceed 3,600 mg/day. There are more conservative options for dose progression, for example: 300 mg/day in week one, 600 mg/day in week two, 900 mg/day in week three, followed by biweekly or even monthly increases up to the total dose of 1,800 mg/day.

Pregabalin: the initial dose is 150 mg/day divided into two doses (two 75 mg tablets/day). It is usual to follow a progressive regimen: 75 mg on day one, 150 mg on day two. From then on, it is possible to progress in increments of 75 mg/day every three days up to a total dose of 450 mg/day. Data associated with significant improvement above this dose are lacking. The limit of 600 mg/day should not be exceeded. There are also more conservative options for dose progression, for example: 50-75 mg/day in week one, 100-150 mg/day in week two, 150 mg/day in week three, followed by weekly or even biweekly increases up to a total dose of 450 mg/day.

### Side effects

Although gabapentinoids are generally well tolerated, they are not free from side effects. Common adverse effects include dizziness, drowsiness, peripheral edema, weight gain, dry mouth, nausea, blurred vision, and mood changes. Pregabalin is associated with a lower risk of adverse effects compared with gabapentin due to its more predictable pharmacokinetics. Gabapentinoids rarely cause serious side effects such as angioedema and respiratory depression, especially at high doses or when combined with other CNS depressants.

### Risks and precautions

1. Suicidal thoughts: as with many drugs that affect the CNS, some people, especially children, adolescents, and young adults, may experience an increase in suicidal thoughts or behavior when taking gabapentinoids. Close monitoring and regular communication with a healthcare professional are important during the first few weeks of treatment.
2. Withdrawal symptoms: abruptly stopping gabapentinoids may lead to withdrawal symptoms, including anxiety, insomnia, nausea, and sweating. Gradually reducing your medication under the guidance of a healthcare professional may help mitigate the effects of withdrawal.

### Drug interactions

1. Opioids: Combining gabapentinoids with opioids, such as hydrocodone or oxycodone, may increase the risk of respiratory depression and overdose. This combination has been associated with an increased risk of opioid-related deaths.
2. Alcohol and sedatives: combining gabapentinoids with alcohol or other CNS depressants (e.g., benzodiazepines) may increase sedation and impair cognitive and motor skills.
3. Antacids: if taken simultaneously, antacids containing aluminum or magnesium may reduce the absorption of gabapentinoids. It is advisable to take these medications at least two hours apart.
4. Antiepileptic medications: Some antiepileptic medications such as phenytoin or carbamazepine may reduce the effectiveness of gabapentinoids and require dosage adjustments.
5. CYP450 interactions: although gabapentinoids themselves do not significantly interact with CYP450, other drugs that interact with CYP450 enzymes may affect gabapentinoid metabolism.

## Cannabinoids – when and how to use them?

### Description

Cannabinoids, both natural and synthetic, have been gaining attention for their potential analgesic effect in the treatment of chronic pain.^(19,20)^ Although it may be somewhat premature to include this class of drugs in this statement, it seems pertinent in view of the great demand observed in clinical practice and the widespread use that has been perceived in the search for the use of natural cannabis and products containing cannabinoids, as well as the increase in medical prescriptions for this purpose. The International Association for the Study of Pain (IASP), one of the world’s leading entities in the area of expertise, published a position statement in 2021^(21)^ recognizing the legitimacy of the life experience of people who report improvement in their pain with the use of cannabis and cannabinoids, but making it clear that the entity does not endorse their use until rigorous investigations and robust results clearly show the benefits and risks of use in humans.

### Mechanisms of action

Cannabinoids exert their analgesic effects through the endocannabinoid system. Key mechanisms include:

1. Cannabinoid receptor (CB1) activation: CB1 receptors in the CNS modulate pain perception by inhibiting the release of neurotransmitters involved in pain signaling.
2. CB2 receptor activation: CB2 receptors found primarily in the immune system may reduce inflammation, which is often a component of chronic and neuropathic pain.
3. Endocannabinoid release: cannabinoids may increase the activity of endocannabinoids, such as anandamide and 2-arachidonoyl glycerol (2-AG), which play roles in pain modulation.

### Efficacy in chronic pain

Clinical trials and observational studies have shown mixed results regarding the efficacy of cannabinoids in the management of chronic pain. Some people report significant pain relief, while others do not experience the same benefits. Variability in individual responses and in the specific type and dose of cannabinoids used may contribute to these discrepancies.^(22)^

### Dosage recommendations

Cannabinoids are generally safe.^(23)^ However, to date, in the chronic pelvic pain world, there is no robust literature to support this statement to recommend a defined dose or safety margin for the population. Use outside of controlled settings should be discouraged until sufficient data support their use. Practitioners and patients need to be aware that there is evidence of the potential harmful effects of this class of medications for some individuals, particularly adolescents and those with psychiatric disorders.^(24)^ Another important issue is the legal status of derivatives, which varies widely between jurisdictions. Healthcare professionals should be aware of local regulations and prescribe cannabinoids in accordance with the law.^(25)^

### Side effects

The use of cannabinoids is associated with several side effects, including dizziness, dry mouth, increased appetite, sedation, and cognitive impairment, impairing coordination and reaction times. In addition, there are concerns about the potential for dependence and withdrawal. It is essential to consider the patient’s overall health, potential drug interactions, and individual response when using cannabinoids for pain management.

### Risks and precautions

1. Psychosis and anxiety: In some individuals, especially those with a predisposition to psychiatric conditions, cannabinoids may trigger or worsen psychotic symptoms or anxiety.
2. Paranoia: Some individuals may experience heightened feelings of paranoia or anxiety when using cannabinoids.
3. Impairment of memory and cognitive function: Long-term use of cannabinoids, especially at high doses, may affect memory, attention, and cognitive function.
4. Cardiovascular effects: cannabinoids can cause changes in heart rate and blood pressure, which can be a concern for people with heart problems.
5. Respiratory effects: smoking cannabis can lead to respiratory problems similar to those caused by tobacco, including chronic bronchitis and lung problems.
6. Dependence: Long-term use of cannabinoids can lead to physical and psychological dependence in some people.
7. Withdrawal syndrome: stopping cannabinoids after regular use can result in withdrawal symptoms, including irritability, insomnia, loss of appetite, and mood swings.

### Drug interactions

1. Alcohol: Combining cannabinoids with alcohol may increase the risk of drowsiness, impaired coordination, and cognitive impairment.
2. CNS depressants: Combining cannabinoids with other CNS depressants, such as benzodiazepines, opioids or sedative medications may potentiate the sedative effects and increase the risk of respiratory depression.
3. Anticoagulants: some cannabinoids, such as THC, may interact with anticoagulants, potentially affecting their anticoagulant effects.
4. CYP450 interactions: certain cannabinoids may interact with the enzyme system. This may affect the metabolism and efficacy of other medications.
5. Antipsychotic medications: combining cannabinoids with antipsychotic medications may result in increased sedation and cognitive impairment.
6. MAOIs: combining cannabinoids with MAOIs may result in unpredictable effects and is generally not recommended.

## Final considerations

Based on the best current evidence, we recommend the following as first-line treatment for neuropathic and nociplastic pain, in order of priority:

Neuropathic:

1. Gabapentinoid
2. Tricyclic antidepressant
3. SNRI antidepressant

Nociplastic:

1. SNRI antidepressant
2. Gabapentinoid
3. Tricyclic antidepressant
